# Pilot trial of a group cognitive behavioural therapy program for comorbid depression and obesity

**DOI:** 10.1186/s40359-020-00400-w

**Published:** 2020-04-17

**Authors:** Taryn Lores, Michael Musker, Kathryn Collins, Anne Burke, Seth W. Perry, Ma-Li Wong, Julio Licinio

**Affiliations:** 1 South Australian Health and Medical Research Institute, Adelaide, SA 5001 Australia; 2 CALHN, Royal Adelaide Hospital, Adelaide, SA 5000 Australia; 3 College of Medicine, SUNY Upstate Medical University, Syracuse, NY 13210 USA

**Keywords:** Depression, Obesity, Comorbid, Cognitive behavioural therapy, CBT, Psychotherapy, Emotional health

## Abstract

**Background:**

Depression and obesity are significant global health concerns that commonly occur together. An integrated group cognitive behavioural therapy program was therefore developed to simultaneously address comorbid depression and obesity.

**Methods:**

Twenty-four participants (63% women, mean age 46 years) who screened positively for depression with a body mass index ≥25 were recruited from a self-referred general population sample. The group therapy program (10 two-hour weekly sessions) was examined in a single-arm, before-after pilot trial, conducted in a behavioural health clinic in Adelaide, Australia. Primary outcomes included survey and assessment-based analyses of depression, anxiety, body image, self-esteem, and weight (kg), assessed at four time-points: baseline, post-intervention, three-months and 12-months post program. Eighteen participants (75%) completed the program and all assessments.

**Results:**

Significant improvements in depression, anxiety, self-esteem and body shape concern scores, several quality of life domains, eating behaviours and total physical activity (among others) – but not weight – were observed over the course of the trial.

**Conclusions:**

Results from this pilot trial suggest that combining interventions for depression and obesity may be useful. Further development of the program, particularly regarding the potential for physical health benefits, and a randomised controlled trial, are warranted.

**Trial registration:**

Trial registration: ANZCTR, ACTRN12617001079336, 13 July 2017. Retrospectively registered after date of the first consent (6 July 2017), but before the date of the first intervention session (20 July 2017).

## Background

Depression and obesity are significant global health concerns. Depression is recognised as the single greatest contributor to global disability, accounting for 7.5% of all YLD (Years Lived with Disability), and afflicting over 300 million people worldwide [1]. One million Australian adults experience depression each year, with an estimated one in six people being diagnosed at some point during their lifetime [2]. People with depression are at increased risk of developing other mental health problems such as anxiety, substance abuse and other mood disorders [3]. Interpersonal relationships are often impaired, leading to isolation, reduced social and emotional health, and decreased productivity. Depression can also significantly impact physical health and is associated with reduced immune function and increased incidence of cardiovascular disease, type 2 diabetes mellitus, stroke and Parkinson’s disease, among others [4–7]. Weight gain is commonly experienced, and as outlined below, there are clear reciprocal links between depression and being overweight or obese [8].

Obesity is typically assessed using the Body Mass Index [BMI = weight (kg)/height (m)^2^] (although more precise definitions of obesity may be preferred at the individual level [9]). A BMI of 25–29.9 is classified as overweight, and a BMI ≥ 30 as obese [10]. Obesity rates have increased substantially over the past several decades. Current estimates suggest that nearly 30% of the world’s population is overweight or obese, with even higher numbers in developed countries such as in Australia (64%) and the United States (68%) [11, 12]. For ease of reference, herein “obese” or “obesity” will comprise both clinical categories (overweight and obesity, i.e. BMI ≥ 25). Explanations for obesity’s rising prevalence include sedentary lifestyles and poor diets, combined with biological, genetic and psychological vulnerabilities [8, 13–15]. As with depression, obesity is associated with greater risk of health problems including cardiovascular disease, type 2 diabetes mellitus, stroke, high blood pressure and certain cancers [16, 17]. Obesity can also negatively impact a range of psychosocial factors such as sociability, willingness to exercise, body image and self-esteem [15].

Separately, depression and obesity are significant problems, but both may be most refractory when comorbid, as commonly occurs [18, 19]. Obesity at baseline has been associated with a 1.55-fold increase in depression incidence at follow-up, and depression at baseline has been associated with a 1.58-fold increased risk of developing obesity [8, 20]. This reciprocal relationship may be due to multiple biological, cognitive and/or behavioural feedback loops that likely drive the pathology of both conditions such that obesity begets depression, leading to greater obesity (and vice versa) [19, 21]. Both are evidenced to be multigenic and multifactorial disorders. Underlying biological mechanisms common to both depression and obesity include, at minimum, endocrine and hypothalamic–pituitary–adrenal (HPA) axis dysregulation; neuroimmune and neuroinflammatory pathways; metabolic, bioenergetic, and oxidative stress pathways; neurogenesis and neuroplasticity; and neurotransmitter disruptions [19, 21, 22]. Behaviourally, a person who experiences depression may reduce their activity and increase their intake of comfort foods, resulting in weight gain and elevated cortisol, which in turn promotes appetite (especially for less healthy foods) and further weight gain [19, 21, 22]. Antidepressants may also be associated with increased appetite and weight gain, particularly in the context of unhealthy lifestyles [23, 24]. Cognitively, negative thinking patterns and low self-worth contribute to distress and unhelpful behavioural responses. Likewise, a person struggling with obesity may experience body dissatisfaction and social stigma, which may lead to distress, reduced self-esteem and depression, particularly when these symptoms are endured over long periods. Thus, these reciprocal links act to mutually reinforce both conditions [19, 21].

For these reasons, there is significant need for integrated therapies that can effectively treat depression and obesity together. Here we present a preliminary quasi-experimental (single-arm) before-after pilot trial of a newly developed group-based psychological intervention program for people with depression and comorbid obesity, that incorporates elements of both cognitive behavioural therapy (CBT) as well as some mindfulness and acceptance techniques/strategies. We hypothesised that participants completing this novel group psychological intervention program would exhibit 1) reduced levels of depression and anxiety (i.e. better mental health), 2) increased healthy eating habits, physical activity, sleep quality and stress management capacity (i.e. more health-positive behaviours), 3) fewer weight-related negative cognitions (i.e. increased self-esteem and body image positivity), and 4) weight loss, at post-intervention and follow-up. The results of this pilot trial and implications for further development of this program and integrative treatment of obesity comorbid with depression are discussed.

## Methods

### Group therapy program protocol

Other studies have demonstrated the effectiveness of psychological therapies for the treatment of depression [25] and obesity (weight loss) [26] independently, but few studies have endeavoured to treat them concurrently. To fill this gap, we developed a novel group therapy program for simultaneously treating comorbid depression and obesity within a single unified psychological intervention. The protocol incorporated a range of cognitive-behavioural, acceptance and mindfulness strategies that have been shown to be efficacious in the treatment of both depression and obesity. The resulting program consisted of 10 two-hour group sessions held weekly at the South Australian Health and Medical Research Institute (SAHMRI), co-facilitated by a psychologist and a mental health professional. Each session focused on a different topic, included reinforcement of prior learning and allocated home-based practice. Key topics covered in the program are outlined in supplemental eTable 1.

### Ethical considerations and trial registration

Ethics approval was granted by the Flinders University Social and Behavioural Research Ethics Committee (SBREC) in May 2017 (Project No. 7601), and the trial was retrospectively registered with the Australian New Zealand Clinical Trials Registry (ANZCTR) (http://www.anzctr.org.au) (registration submitted 13 July 2017; registration recorded 25 July 2017; trial number ACTRN12617001079336), after the date of the first consent (6 July 2017), but before the date of the first intervention treatment session (20 July 2017). The full trial protocol is available here: (https://anzctr.org.au/Trial/Registration/TrialReview.aspx?ACTRN=12617001079336). All participants were provided with a participation information sheet and consent form prior to interview, and informed written consent was obtained for study participation. All research at SAHMRI adheres to the Australian Government National Statement on Ethical Conduct in Human Research (2007), which details sections on Scope, Methods, Recruitment, Collection and management of data and dissemination of results [27].

### Sampling and trial design

Participants were recruited from the general population in Adelaide, South Australia by advertisement of the study on local radio, the SAHMRI website and online news media over a period of 3 months. Interested individuals were directed to a SAHMRI website to register, after which they were contacted by phone for a brief screen with one of the primary researchers. During the telephone screening, potential participants were provided with an overview of the program, key trial aims, and participation details, and they were asked whether they could commit to the 10-week program for the specified dates. Eligibility was then assessed and a face-to-face appointment to complete the initial (baseline) assessment was arranged for those meeting inclusion criteria.

Participants were assessed in four separate appointments at SAHMRI: baseline (i.e. within 2 weeks prior to the start of the group therapy program); immediate post-intervention (within 2 weeks of the program ending); 3 months post-intervention (within 2 weeks of the target date); and 12 months post-intervention (within 2 weeks of the target date). To maintain the small group size required for group therapy [28], two cohorts of participants completed the 10-week program at SAHMRI in a single-arm before-after pilot study design (i.e. all participants were subject to the same intervention), and the results for all participants who completed the program were analysed together. Completion of the program required participants to attend at least seven of the ten sessions (70% attendance), in order for the effectiveness of the program to be properly evaluated [29]. Consistent with a per protocol analysis, those who did not meet this criterion (70% attendance) were excluded from analysis. The first group completed the 10-week program in July – September 2017, and the second group in January – March 2018.

### Participants

Adults aged 18–65 years, with BMI ≥ 25 (i.e. overweight or obese) and scoring ≥5 on the Patient Health Questionnaire (PHQ-9 – depression screening tool) [30] were eligible to participate in the trial. Individuals with a major systemic or physical illness that affected weight; an uncontrolled thyroid disorder; an eating disorder diagnosed within the last twelve months; an uncontrolled psychiatric or personality disorder; a problematic use of alcohol or other substances; or intellectual impairment or other cognitive deficits that could impair progress in the group setting were excluded. Antidepressant medication use was allowed, however concurrent participation in other forms of psychological therapy for depression or obesity/weight management was not.

### Measures

#### Sample characteristics

Demographic data (including age, sex, and cultural background) were collected via an initial survey at the first assessment appointment. Use of antidepressant medication and presence of comorbid physical health problems were also recorded.

#### Primary outcome measures

A range of primary outcome measures were administered. Specifically, depression severity was measured with the Hamilton Depression Scale (HAM-D), anxiety with the Hamilton Anxiety Scale (HAM-A), self-esteem with the Rosenberg Self-Esteem Scale (RSES), and body image concerns with the Body Shape Questionnaire (BSQ-34). Participant weight (kg) and height (cm) were collected and BMI was calculated (kg/m^2^).

#### Secondary outcomes measures

Health-related quality of life was assessed by the RAND-36 item Health Survey (SF-36), which measures quality of life in eight domains, including physical functioning, emotional wellbeing and general health. Physical activity (including number of days, hours or minutes of total, vigorous, moderate, and sedentary activity) was gauged with the Global Physical Activity Questionnaire (GPAQ). Nutritional intake was assessed with the Commonwealth Scientific and Industrial Research Organisation (CSIRO) Healthy Diet Score questionnaire, which provides a total overall diet quality score as well as number of daily servings of different types of food and beverages (e.g. fruits and vegetables, discretionary foods). Additional health behaviours measured included eating behaviour (for example, restrictive and emotional eating) with the Three Factor Eating Questionnaire (TFEQ-R18), alcohol consumption with the Alcohol Use Disorders Identification Test (AUDIT), and sleep quality (including various sleep components) with the Pittsburgh Sleep Quality Index (PSQI).

Additional physical measurements were also collected: specifically, waist circumference (cm), hip circumference (cm), blood pressure (systolic and diastolic mm Hg), and pulse rate (beats per minute). Waist-to-hip and waist-to-height ratios were calculated. Participants were also asked to rate their current level of mental health and physical health, as well as their readiness to change and confidence in making the changes required to improve their health.

Primary and secondary outcome measures are described in greater detail in the Supplemental materials. Score ranges and clinical interpretations are displayed in Table 2.

### Evaluation of treatment acceptability

At the end of their 10-week group therapy program, participants completed a short evaluation form which asked them to rate their experience of the intervention across 22 items (e.g., each session’s quality, early experiences in the program, location, timing, group dynamics) on a 5-point scale from 1 (very poor) to 5 (very good).

### Statistical analysis

Recommended minimum sample sizes for pilot studies of this nature, which are in themselves intended to determine sample sizes required to achieve appropriate statistical power and suitability for future randomised controlled trials (RCTs), is *n* = 12 [31], plus 15% for non-parametric tests [32] (i.e. *n* = 14). Our sample size exceeded that value and provided adequate power. Descriptive statistics and frequencies were generated on baseline data to describe the characteristics of the population at intake. Variables at all four time-points were inspected for outliers and distribution characteristics to check assumptions for conducting repeated measures analyses. Several variables violated assumptions of normality and outliers. Therefore, the non-parametric Friedman test was used to investigate changes in outcomes over the four time-points (baseline, post-intervention, 3-month follow-up and 12-month follow-up): *p* < .05 was considered significant. Follow-up pairwise comparisons were performed to determine where the significant changes occurred.

## Results

### Sample characteristics at baseline

**Fig. 1:**
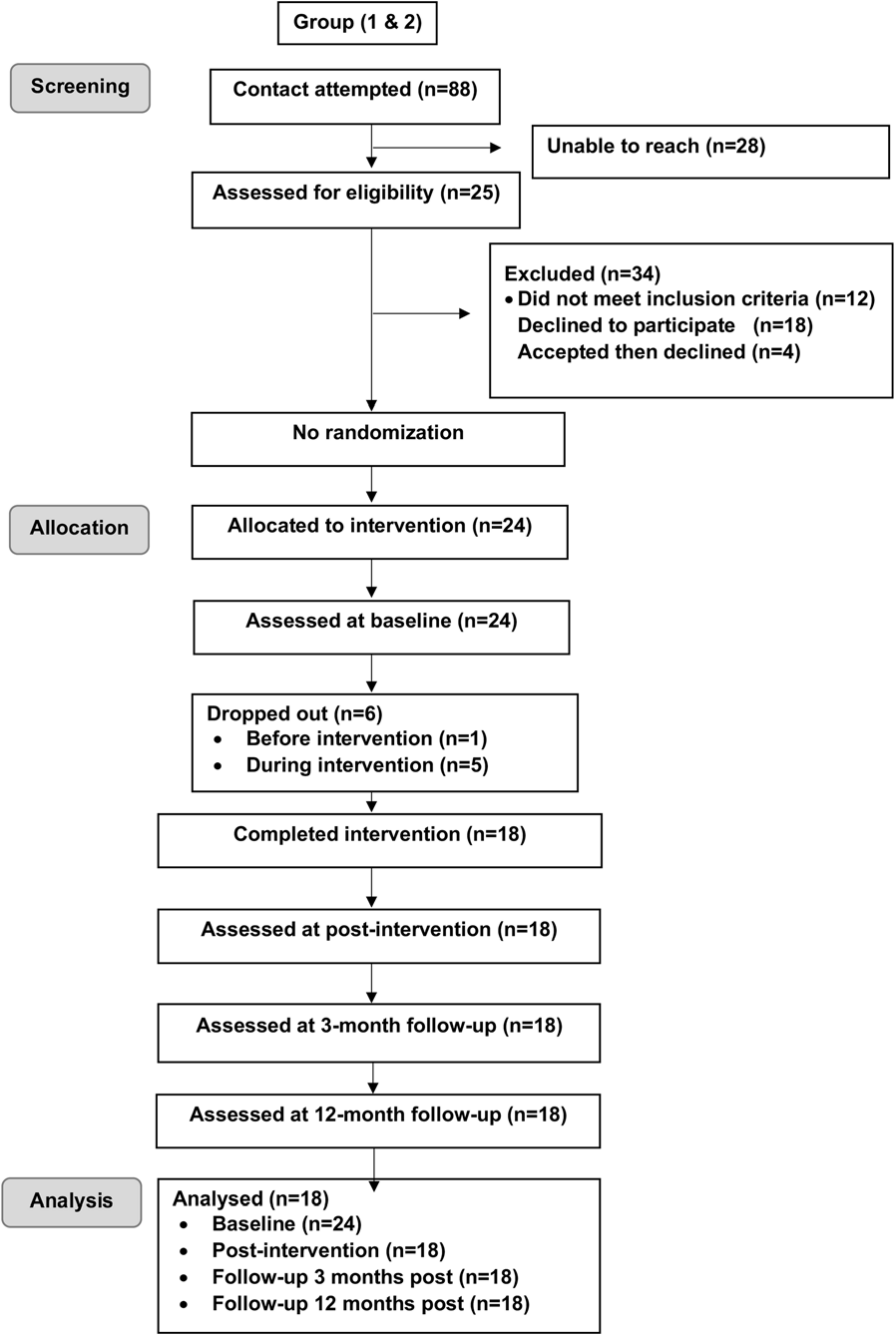
CONSORT diagram depicting flow of participants through trial

The CONSORT-type diagram for flow of participants through the trial is shown in Fig. 1. A total of 24 participants were recruited to the program across two pilot groups. Of these, 18 completed the program, attending an average of 8.8 out of the 10 sessions. Three individuals provided reasons for declining/ceasing the program (“too many commitments” – dropped out before session one, “not comfortable with other group members” – dropped out after session one, and “dealing with a relationship break-up” – dropped out after session five). The remaining three participants did not respond to requests for a withdrawal reason. Sample characteristics of participants are shown in Table 1.

**Table 1:** Sample characteristics of trial sample at baseline (*n* = 23) ^a^

	**Count (%)**
Sex (*n* = 24)	
Women	15 (62.5%)
Men	9 (37.5%)
Relationship status	
Single / never married	5 (20.8%)
Divorced / separated	3 (12.5%)
Married / de facto	15 (62.5%)
Antidepressant use (current)	15 (62.5%)
Physical health comorbidities	9 (38%)
Smoking status	
Current smoker	1 (4.2%)
Ex-smoker	9 (37.5%)
Non-smoker	13 (54.2%)
Ethnicity	
Caucasian	21 (87.5%)
Aboriginal / TSI	1 (4.2%)
Indian	1 (4.2%)
Education	
Some school	3 (12.5%)
High school graduate	3 (12.5%)
Technical / vocational training	8 (33.3%)
Bachelor’s degree	6 (25%)
Post-graduate degree	3 (12.5%)
Employment status	
Homemaker	3 (12.5%)
Self-employed	3 (12.5%)
Salaried income	11 (45.8%)
Unable to work	3 (12.5%)
Retired	1 (4.2%)
Studying	2 (8.3%)
Age (mean years)	^b^46.39 (10.08), range 18–64

^a^NB: demographic data (except for sex) missing for one participant

^b^NB: Refers to M (SD), all other data refer to n(%)

### Baseline (pre-intervention) assessment

Prior to starting the program, the 24 initial participants (i.e. pre-dropouts) reported moderate to severe levels of depression, mild to moderate levels of anxiety, normal self-esteem, and moderate concerns with body shape. On average, participants’ BMI was within the moderately obese range (obese class II). Their mean score on the CSIRO Healthy Diet questionnaire was below the Australian national average of 59, and their reported average daily servings of fruit and vegetables were below national recommendations. Participants were not meeting the recommended amount of physical activity per week, and sleep quality was poor. Table 2 displays participants’ mean scores at baseline for primary (and some secondary) outcome variables.

**Table 2:** Mean outcome scores of trial sample at baseline (*n* = 24)

	M	(SD)	[95% CI]	(SE)	[Omega]	Score Range	Clinical Interpretation
**Psychological measures**							
Depression (HAM-D)	17.83	(6.59)	[15.05–20.61]	(1.34)	[0.80]	0–52	> 17 moderate to severe
Anxiety (HAM-A)	15.96	(8.99)	[12.16–19.75]	(1.83)	[0.85]	0–56	18–24 mild to moderate
Self-esteem (RSES)	22.98	(5.92)	[20.48–25.48]	(1.21)	[0.92]	0–30	15–25 normal
Body image concerns (BSQ-34)	113.92	(34.53)	[99.34–128.5]	(7.05)	[0.97]	34–204	111–140 moderate
**Physical measurements**							
Weight (kg)	111.5 kg	(18.50)	[103.7–119.3]	(3.78)			
BMI (kg/m2)	39.39	(7.74)	[36.11–42.66]	(1.58)			> 30 obese - class II
Waist-to-height ratio (cm/cm)	.71	(.08)	[.67–.74]	(.02)			>.63 for men, >.58 for women - morbidly obese
**Health behaviours**							
CSIRO diet score (CSIRO)	47.75	(12.55)	[42.32–53.18]	(2.61)		1–100	below national average of 59
Vegetable intake^a^ (CSIRO)	3.29	(3.41)	[1.81–4.76]	(0.71)			< recommended 5
Fruit intake^a^ (CSIRO)	1.14	(1.25)	[.60–1.68]	(0.26)			< recommended 2
Total physical activity^b^ (GPAQ)	2.10	(3.07)	[.80–3.39]	(0.63)	[0.78]		< recommended 2.5
Overall sleep problems (PSQI)	9.33	(3.91)	[7.39–11.28]	(0.92)	(0.76)	0–21	> 5 poor sleep quality

^a^ daily servings; ^b^ hours per week of moderate + physical activity. Abbreviations: *HAM-D* Hamilton Depression Rating Scale, *HAM-A* Hamilton Anxiety Rating Scale, *RSES* Rosenberg Self-Esteem Scale, *BSQ-34* Body Shape Questionnaire, *CSIRO* Commonwealth Scientific and Industrial Research Organisation, *GPAQ* Global Physical Activity Questionnaire, *PSQI* Pittsburgh Sleep Quality Index. Statistics: M, SD, CI, SE, Omega are, respectively, Median, Standard Deviation, 95% Confidence Interval, Standard Error, and McDonald’s Omega (ω)

Baseline scores for additional secondary outcome variables (e.g. participants’ subjective ratings of their own mental and physical health, readiness to change, and confidence to make the changes required, as well as other physical, quality of life, and health behaviour data) are summarised in supplemental eTable 2, for all 24 subjects prior to the start of the program (pre-dropouts).

### Program outcomes

**Fig. 2:**
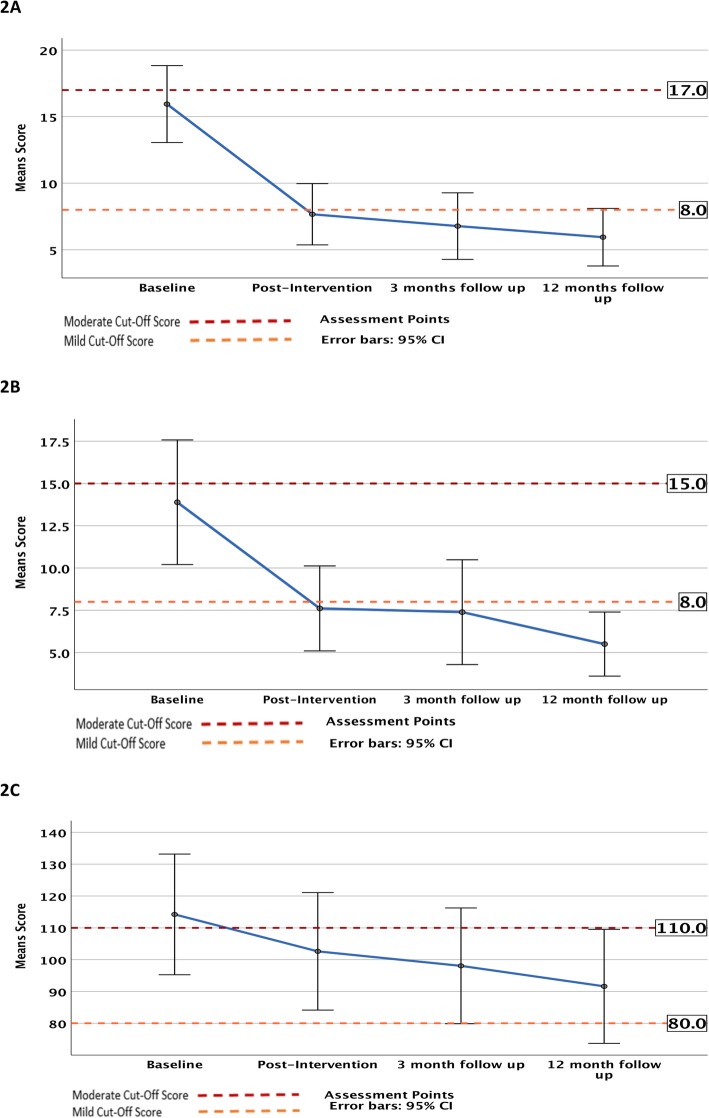
Changes in median scores for trial sample over time. **a** Changes in depression scores over time. **b** Changes in anxiety scores over time. **c** Changes in body shape concern scores over time

There were significant changes for multiple primary and secondary outcome measures over the course of the trial, including participant self-ratings of health, depression, anxiety, body shape concerns, self-esteem, several dimensions of quality of life and two types of health behaviours (Table 3). Specifically, participants’ depression scores declined significantly between baseline and post-intervention, and this improvement was maintained at three-month and 12-month follow-ups (*p* = .001, <.001, and < .001, respectively). Likewise, there was a significant decrease in anxiety scores between baseline and post-intervention (*p =* .049), with the improvement remaining significant at both three-month (*p* = .004) and 12-month follow-up (*p* < .001). Participants’ body shape concerns declined over the course of the trial: the change between baseline and post-intervention was not significant, but the reduction from baseline was statistically significant at three-month(*p* = .018) and 12-month follow-up(*p* < .001). These three notable findings are illustrated in Fig. 2. Self-esteem increased over the trial period, but the change was only statistically significant between baseline and 12-month follow-up (*p* = .006).

**Table 3:** Changes in medians of trial sample over time (outcomes from Friedman test) (*n* = 18)

	Baseline	Post-Intervention	3 Month Follow-Up	12 Month Follow- Up					
	Median	Median	Median	Median	Range	X^**2**^	df	***p***-value	η_**ρ**_^**2**^
**Subjective ratings**									
Mental health	5	6	6.5	7.5	1–10	14.86	3	0.002	0.304
Physical health	3.5	5	4	6	1–10	11.3	3	0.01	0.217
Psychological measurements									
Depression (HAM-D)	17	6.5	5	5.5	0–52	29.8	3	<.001	0.62
Anxiety (HAM-A)	15	6	5.5	4.5	0–56	24.09	3	<.001	0.507
Self-esteem (RSES)	22.5	27	26.5	26.75	0–30	11.63	3	0.009	0.201
Body shape concerns (BSQ-34)	117	108.5	102	94	34–204	22.23	3	<.001	0.388
**Physical measurements**									
Weight (kg)	109.2	109.15	109.55	109.55		2.07	3	0.557	0.042
BMI (kg/m^2^)	36.97	37.79	37.37	37.11		1.86	3	0.602	0.039
Quality of life (SF-36)									
Role limitations physical	62.5	62.5	75	100	0–100	8.48	3	0.037	0.105
Role limitations emotional	0	33.33	33.33	50	0–100	15.62	3	0.001	0.273
Energy / fatigue	15	15	30	42.5	0–100	16.13	3	<.001	0.519
Emotional wellbeing	44	44	68	74	0–100	32.47	3	<.001	0.57
General health	30	37.5	35	45	0–100	18.56	3	<.001	0.318
**Health Behaviours**									
CSIRO diet score (CSIRO)	51.1	52.3	49.3	50.3	0–100	3.36	3	0.34	0.044
Discretionary food intake^a^ (CSIRO)	6.12	3.61	3.56	2.73		6.66	3	0.084	0.292
Emotional eating (TFEQ-R18)	89	66.67	66.67	66.67	0–100	14.24	3	0.003	0.27
Uncontrolled eating (TFEQ-R18)	57.41	40.74	51.85	40.74	0–100	5.71	3	0.127	0.216
Cognitive restraint (TFEQ-R18)	27.78	38.89	36.11	47.22	0–100	21.47	3	<.001	0.344
Total physical activity^b^ (GPAQ)	1.25	2.25	1.13	3.65		8.56	3	0.036	0.152
Sedentary activity^b^ (GPAQ)	80.5	84	75.3	71.5		4.15	3	0.246	0.071
Overall sleep problems (PSQI)	10	7	9	8.5	0–21	2.74	3	0.434	0.086

^a^ daily servings; ^b^ hours per week Abbreviations: *HAM-D* Hamilton Depression Rating Scale, *HAM-A* Hamilton Anxiety Rating Scale, *RSES* Rosenberg Self-Esteem Scale, *BSQ-34* Body Shape Questionnaire, *CSIRO* Commonwealth Scientific and Industrial Research Organisation, *GPAQ* Global Physical Activity Questionnaire, *PSQI* Pittsburgh Sleep Quality Index, *SF36* RAND 36-Item Health Survey 1.0; *TFEQ-18* Three Factor Eating Questionnaire – Revised, *η*_*ρ*_^*2*^ partial Eta Squared

Participants’ self-rated mental health did not change significantly between baseline and post-intervention but was significantly improved at three-month and 12-month follow-up (*p* = .040 and .027, respectively). Self-rated physical health only improved between baseline and 12-month follow-up (*p* = .018). With respect to dimensions of quality of life, participants’ emotional wellbeing showed no change at the end of the program but was improved versus baseline at both three-month (*p* = .032) and 12-month follow-up (*p* < .001). Similarly, energy/fatigue showed no change post-intervention, but was improved versus baseline at three-month and 12-month follow-up (*p* = .039 and < .001, respectively). General health improved between baseline and post-intervention (*p* = .030), and this was maintained at 12-month follow-up (*p* = .001). Participants’ role limitations due to physical health problems significantly improved over the course of the study; however, post-hoc comparisons were non-significant. Role limitations due to emotional problems also improved, but only between baseline and 12-month follow-up (*p* = .003).

Regarding health behaviours, emotional eating behaviour decreased between baseline and post-intervention (*p* = .022) and remained significantly reduced 12 months later (*p* = .018). Cognitive restraint did not change post-intervention or at three-month follow-up but had increased at 12-month follow-up relative to baseline (*p* < .001). Participants’ total hours of physical activity per week significantly increased (*p* = .036), most notably between baseline and 12-month follow-up (*p* = .010).

Additional secondary and non-significant (*p* > .05) outcomes are shown in eTable 3 in the Supplemental materials. Other domains of quality of life and numerous health behaviours did not change throughout the trial. There were no significant changes in participants’ weight or other physical measurements (e.g. waist-to-height ratio) between baseline and post-intervention or at three-month or 12-month follow-up. Moreover, there were no significant changes in participants’ nutritional intake or sleep. However, the results of preliminary analyses conducted immediately post-intervention showed significant improvements between baseline and post-intervention in uncontrolled eating behaviour, discretionary food intake, and overall sleep problems – see eTable 4 in Supplemental materials – but these changes were not statistically significant when the comparison was made across all four time-points in the main analysis (Table 3).

### Evaluation of treatment acceptability

Quantitative feedback gathered from participants at the end of the final session of the group therapy program was predominantly positive with the majority providing ratings of “good” to “very good” (eTable 5). The open-ended qualitative feedback was also encouraging (refer to eTable 6 for a sample). Participants were further given the opportunity to provide verbal feedback to the other group members and facilitators during the final session, and this was overwhelmingly positive.

## Discussion

This pilot study sought to examine the potential benefits of a newly developed group-based psychological intervention program for people with comorbid depression and obesity. The main aims of the program were to help people achieve better mental health, establish and/or increase health behaviours, improve weight-related cognitions, and facilitate weight loss. We also hoped that focusing on overall mind and body health would lead to improvements that were sustainable over the medium to long-term. We found there was a significant reduction in participants’ depression scores by program-end, with many moving from the moderate/severe to normal (non-clinical) ranges on the Hamilton Depression Scale (HAM-D). Additionally, participants’ HAM-A anxiety scores also decreased significantly, moving from the mild/moderate to normal ranges. Importantly, improvements in both variables were maintained at follow-up 3 months and 12 months after the intervention had ended. The group therapy program therefore has considerable potential to be effective in helping people enjoy better mental health – one of its key aims.

There is also some evidence of improvements in weight-related negative cognitions, with participants experiencing a significant reduction in body shape concerns by 3 months post-program (moving from moderate to mild levels on the BSQ-34) – an improvement which was maintained 12 months after the program ended. For the target population, improving poor body image is crucial, given its potential to be a trigger for unhelpful thoughts and unhealthy eating behaviours. Our finding that body shape concerns were significantly improved at both follow-up time-points (versus baseline), but not immediately post-intervention, suggests that changing perceptions about one’s body is a longer process than the 10-week duration of the program. The same may be true for shifting self-esteem, which demonstrated an upward trend over time, and was significantly improved at 12 months after the program ended.

Several other results support the notion that continued improvement in program outcomes may be seen over time. For example, several domains of quality of life (e.g. emotional wellbeing and energy/fatigue) improved only at follow-up (at three and 12 months) but not immediately post-program. For many individuals, depression and obesity are complex and chronic, so it makes sense that a longer period for further practice and reinforcement may be necessary for measurable changes to occur. That said, the degree to which participants in our trial continued practicing strategies or adhered closely to their individual goals after post intervention is unknown. Future studies could investigate ideal program duration for this approach, and whether ongoing monitoring or follow-up sessions could enhance outcomes.

Significant improvements were seen in some health behaviours (emotional eating, cognitive restraint, and total physical activity; Table 3), and trends toward improvements seen in others (uncontrolled eating [*p* = .127] and discretionary food intake [*p* = .084]). Physical activity, nutrition, and eating behaviours are associated with both physical and mental health, hence the importance of establishing and strengthening healthy lifestyle habits in this target population. The program could be further developed to build on these improvements, including perhaps involvement and co-facilitation by a dietician and/or exercise physiologist.

Participants did not experience significant weight loss immediately post-intervention or at follow-up assessments three and 12 months later. A potentially confounding variable that may prevent weight loss (particularly when accompanied by an unhealthy lifestyle) is antidepressant therapy [23, 24], and 11 of our 18 subjects were taking antidepressants during the program. While our current trial was not designed nor powered to answer the question of whether antidepressant status affects weight loss during/after the intervention, larger future trials can incorporate this question into their design. Preliminary analyses do indicate, however, that improvements in depression scores are similar even if cohorts are subdivided into medicated versus un-medicated groups; that is, antidepressant status does not appear to be responsible for the improvements in depression scores. However, further validation in a larger study is required. An active dietary intervention is also likely to be needed if weight loss is the main desired outcome.

The results of our pilot trial are similar to those reported in other recent investigations of combined treatment approaches. For example, one recent RCT of 1025 individuals [33] found that a psychological therapy focused on food-related behavioural activation was associated with improved anxiety and depression scores in overweight or obese adults over time, particularly when depression severity was higher at baseline (although the treatment approach did not prevent the development of major depressive disorder at 1 year follow-up). Weight loss and body weight perceptions were not reported in this study. In another, the addition of a cognitive behavioural therapy (CBT) component for depression (CBTD) to a behavioural weight control (BWC) program resulted in some weight loss, described as modest but less than that typically seen with non-depressed patients in BWC therapies (discussed in [34]). The combined approach did not significantly augment weight loss versus BWC therapy alone [34, 35]. Compared to our program, the BWC + CBTD treatment incorporated more directive and hands-on measures designed to achieve weight loss (e.g. prescriptions for calorie intake/diet and amount of weekly exercise). Nevertheless, together our and others’ results [33–35] demonstrate both the potential of these combined approaches and the difficulties they inherently face, particularly in achieving large and/or sustained weight loss and other health benefits in depressed individuals. Further work to develop these programs to more effectively achieve these goals is required.

We acknowledge that there are limitations to this pilot trial, the main one being that there was no control arm of ‘usual care’ or healthy controls for comparison purposes. We therefore cannot rule out the possibility that psychological health may have improved naturally over time. Future trials of this program should include a control arm in order to appropriately evaluate efficacy. In addition, a potential confounding factor results from the fact that participants using antidepressants were not excluded from the trial. However, improvements in depression scores were found to be similar for medicated and un-medicated sub-groups. A larger sample size in future trials would also bolster confidence in the results.

## Conclusions

Taken overall, the results of this pilot trial of a novel group therapy program are promising and warrant further development and evaluation of the intervention. The current program targeting comorbid depression and obesity is perceived as useful and acceptable by participants and has potential to improve health outcomes, particularly psychological well-being. Further refinement of the program to include additional content on specific health-positive behaviours and weight loss measures will now be completed. Larger randomised controlled trials are also required to substantiate these preliminary findings and further examine potential improvements in physical health outcomes.

## Supplementary information

**Additional file 1.**

## Data Availability

All data needed to evaluate the conclusions in the paper are present in the paper and/or the Supplementary Materials. Additional data available from authors upon request.

## Abbreviations

ANZCTR: Australian and New Zealand Clinical trials registry
YLD: Years Lived with Disability
BMI: Body Mass Index
SAHMRI: South Australian health and medical research institute
SBREC: Social and behavioural research ethics committee
PHQ-9: Patient Health Questionnaire
HAM-D: The hamilton depression rating scale
HAM-A: Hamilton anxiety rating scale
RSES: Rosenberg self-esteem scale
BSQ-34: Body shape questionnaire
SF-36: RAND 36-item health survey 1.0
GPAQ: Global physical activity questionnaire
CSIRO: Commonwealth scientific and industrial research organisation
TFEQ-R18: Three factor eating questionnaire – revised
AUDIT: Alcohol use disorders identification test
PSQI: Pittsburgh sleep quality index
RCT: Randomised controlled trial
CONSORT: Consolidated standards of reporting trials
CBT: Cognitive behavioural therapy
CBTD: Cognitive behavioural therapy for depression
BWC: Behavioural weight control
