# Supplementary material for: Pilot trial of a group cognitive behavioural therapy program for comorbid depression and obesity
Source: BMC Psychol. 2020 Apr 17;8:34. doi: 10.1186/s40359-020-00400-w (PMC7164235; doi:10.1186/s40359-020-00400-w)
Supplement: Supplementary file 1 — Additional file 1. [file 40359_2020_400_MOESM1_ESM.docx]

**A Group Therapy Program for Comorbid Depression and Obesity**

Taryn Lores, Michael Musker, Kathryn Collins, Anne Burke, Seth W. Perry, Ma-Li Wong, Julio Licinio

**Supplementary Materials**

**Supplementary Methods**

*Measures*

*Baseline data collection*

*Telephone Screening*:

- In the initial telephone screening, potential participants were administered the nine-item *Patient Health Questionnaire* (PHQ-9) ^1^ to screen for depression and determine eligibility for the trial. Items are rated on a 4-point scale and are summed to produce a total score ranging from 0 to 27. Scores between 5–9 are consistent with mild depression, 10–19 moderate depression, and ≥ 20 severe depression.

*Sample characteristics:*

- Participants completed an initial survey to collect demographic information. Variables measured recorded were age, sex, relationship status, education level, and cultural background.
- Antidepressant medication use and presence of comorbid physical health problems were also recorded.

*Primary Outcome Measures*

*Psychological measures:*

- Depression severity was measured using the *Hamilton Depression Rating Scale* (HAM-D) ^2^. This 17-item questionnaire was clinician-rated, with each item scored on 0-2, 3, or 4-point scale, resulting in a total score ranging from 0 to 52. The clinical cut-off score is 8; scores between 0–7 indicate normal functioning, 8–13 mild depression, 14–17 mild to moderate depression, over 17 moderate to severe.
- Anxiety severity was measured using the *Hamilton Anxiety Rating Scale* (HAM-A),^3^ which consists of 14 items clinician-rated on a 4-point scale. The total score range is 0 to 56, with a clinical cut-off of 18. Scores ≤ 17 indicate mild anxiety, 18–24 mild to moderate anxiety, 25–30 moderate to severe anxiety, and ≥ 31 severe anxiety.
- Self-esteem was assessed using the 10-item self-rated *Rosenberg Self-Esteem Scale* (RSES) ^4^. Items are scored on a 4-point scale (1=strongly disagree, 4 = strongly agree). Total scores range from 10 to 40, with higher scores indicating higher levels of self-esteem.

Body image was measured using the 34-item *Body Shape Questionnaire* (BSQ-34) ^5^, which has been applied extensively in both males and females of varied ages and in multiple languages. Items are self-rated on a six-point Likert-type scale. A total score ranges from 34 to 204; scores under 80 are consistent with no concern with body shape, 80–110 indicate mild concern, 111–140 moderate concern, and over 140 marked concern with body shape.

*Physiological measurements:*

- *Participant weight (kg) and height (cm) were collected and BMI was calculated (kg/m^2^).*

*Secondary Outcome Measures*

*Quality of life:*

- The *RAND 36-Item Health Survey 1.0* (SF-36) ^6^ was administered to assess health quality of life. Items are self-rated on 2- and 6-point scales, and are then re-coded (range 0 – 100) and averaged together to generate a total scaled score for 8 domains: physical functioning, bodily pain, role limitations due to physical health problems, role limitations due to personal/emotional problems, emotional well-being, social functioning, energy/fatigue, and general health. Higher scores in each domain indicate a greater state of health.

*Health behaviours:*

- Participants also completed the 16-item *Global Physical Activity Questionnaire* developed by the World Health Organisation (GPAQ) ^7^. Responses are measured in number of days, hours, or minutes, with total scores calculated for four domains: total vigorous activity, total moderate activity, total overall activity, and total sedentary behaviour (across a typical week).
- The (Commonwealth Scientific and Industrial Research Organisation) *CSIRO Healthy Diet Score ^8^* was administered to assess eating habits in relation to Australia's dietary guidelines. This 82-item questionnaire is completed online (https://my.totalwellbeingdiet.com/healthy-diet-score) or on paper, with participants responding to questions about how frequently and in what quantities they consume different types of food and beverages. Responses are scored and analysed by CSIRO software; a total score of overall diet quality is calculated out of 100, with higher scores indicating greater alignment with national guidelines. Average daily servings for individual food groups (e.g. fruit intake, vegetable intake) were also obtained for this study.
- The *Three Factor Eating Questionnaire* – *Revised* (TFEQ-R18) ^9, 10^ was administered to measure eating behaviours. This 18-item self-report measure assesses three types of eating behaviour – cognitive restraint, uncontrolled eating, and emotional eating – with items measured on a 4-point scale. Three scale scores are summed and transformed into a 0-100 scale, with higher values indicating a greater level of the eating behaviour.
- The 10-item *Alcohol Use Disorders Identification Test (AUDIT)* ^11^ developed by the World Health Organisation was used to screen for problematic alcohol use. Questions regarding alcohol consumption, dependence, and alcohol-related problems were scored by participants on a 5-point scale, with a total score calculated and ranging from 0 to 50. Scores 0–7 reflect low risk; 8–15, risky or hazardous alcohol-related behaviours; 16–19, high-risk or harmful levels; and scores ≥ 20 reflect very high-risk alcohol use.
- Finally, the *Pittsburgh Sleep Quality Index* (PSQI)^12^ was used to assesses sleep quality. This questionnaire consists of 19 items, and responses are used to create seven component scores (subjective sleep quality, sleep latency, sleep duration, habitual sleep efficiency, sleep disturbances, use of sleeping medications, and daytime dysfunction) and one global score. A global score (range 0 – 21) of ≥ 5 indicates poor sleep quality.

*Physiological measurements:*

- Waist circumference (cm), hip circumference (cm), blood pressure (systolic and diastolic mm Hg), and pulse rate (beats per minute).
- Waist-to-hip and waist-to-height ratios were calculated.

*Self-ratings:*

- Participants also provided ratings on a 10-point scale (1 = lowest, 10 = highest) of their current level of mental and physical health, as well as their readiness for and confidence in making the changes required to improve their health.

**Supplementary Tables**

| **eTable 1. Overview of program topics** | | |  |
| --- | --- | --- | --- |
| **Session** | **Topic** |  |  |
| 1 | Introduction |  |  |
| 2 | Education |  |  |
| 3 | Movement |  |  |
| 4 | Awareness |  |  |
| 5 | Emotion regulation |  |  |
| 6 | Thinking |  |  |
| 7 | Challenging |  |  |
| 8 | Self-value |  |  |
| 9 | Broader health |  |  |
| 10 | Future directions |  |  |

| **eTable 2. Mean Scores of trial sample at Baseline (secondary outcomes)** (n=24) | | | |
| --- | --- | --- | --- |
|  | ***M* (*SD*)** | **Score range** | **Clinical interpretation** |
| Subjective ratings (out of 10) |  |  |  |
| Mental health | 4.92 (1.84) | 1 – 10 |  |
| Physical health | 3.88 (2.17) | 1 – 10 |  |
| Readiness to change | 8.00 (1.73) | 1 – 10 |  |
| Confidence to change | 7.17 (1.72) | 1 – 10 |  |
| Physical measurements |  |  |  |
| Waist-to-hip ratio (cm/cm) | 0.96 (.08) |  | >.85 for women, >.90 for men – obese |
| Systolic blood pressure (mm Hg) | 131.79 (14.75) |  | 130 – 139 high blood pressure (stage 1) |
| Diastolic blood pressure (mm Hg) | 84.17 (8.64) |  | 80 – 89 high blood pressure (stage 1) |
| Pulse (BPM) | 81.88 (13.23) |  | 60 – 100 normal range |
| Quality of life (SF-36) |  |  |  |
| General health | 35.59 (19.52) | 0 – 100 | Higher scores greater state of health |
| Physical functioning | 65.83 (23.30) | 0 – 100 | Higher scores greater state of health |
| Limitations due to Physical | 42.71 (43.29) | 0 – 100 | Higher scores greater state of health |
| Limitations due to Emotional | 19.44 (29.35) | 0 – 100 | Higher scores greater state of health |
| Emotional wellbeing | 46.50 (17.76) | 0 – 100 | Higher scores greater state of health |
| Energy /fatigue | 17.92 (16.35) | 0 – 100 | Higher scores greater state of health |
| Social functioning | 54.17 (22.92) | 0 – 100 | Higher scores greater state of health |
| Pain | 53.85 (26.52) | 0 – 100 | Higher scores greater state of health |
| Health behaviours |  |  |  |
| Cognitive restraint eating (TFEQ-R18) | 27.47 (17.92 ) | 0 – 100 | Higher scores greater eating behaviour |
| Uncontrolled eating (TFEQ-R18) | 54.73 (22.99) | 0 – 100 | Higher scores greater eating behaviour |
| Emotional eating (TFEQ-R18) | 77.89 (25.29) | 0 – 100 | Higher scores greater eating behaviour |
| Alcohol intake (AUDIT) | 4.54 (4.39) | 0 – 50 | 0 – 7 low risk |

| **eTable 3. Changes in medians of trial sample over time (secondary outcomes; from Friedman test)** (n=18) | | | | | | | | |
| --- | --- | --- | --- | --- | --- | --- | --- | --- |
|  | **Baseline** | **Post-Intervention** | **3 Month Follow-Up** | **12 Month Follow-Up** | **Score range** |  |  |  |
|  | ***Median*** | ***Median*** | ***Median*** | ***Median*** |  | ***X^2^*** | ***df*** | ***p*-value** |
| Self-ratings |  |  |  |  |  |  |  |  |
| Readiness to change | 8.00 | 8.00 | 7.00 | 7.00 | 1 – 10 | 6.21 | 3 | .102 |
| Confidence to change | 8.00 | 7.00 | 8.00 | 8.00 | 1 – 10 | 1.38 | 3 | .711 |
| Physical measurements |  |  |  |  |  |  |  |  |
| Waist-hip ratio (cm/cm) | 0.95 | 0.93 | 0.94 | 0.94 |  | 3.40 | 3 | .334 |
| Waist-height ratio (cm/cm) | 0.71 | 0.69 | 0.71 | 0.67 |  | 9.54 | 3 | .105 |
| Systolic blood pressure (mm Hg) | 132.00 | 126.00 | 134.50 | 128.00 |  | 4.53 | 3 | .210 |
| Diastolic blood pressure (mm Hg) | 83.00 | 83.50 | 81.50 | 81.00 |  | 4.95 | 3 | .175 |
| Pulse (BPM) | 82.00 | 73.00 | 76.00 | 76.00 |  | 3.60 | 3 | .308 |
| Quality of life (SF-36) |  |  |  |  |  |  |  |  |
| Physical functioning | 75.50 | 80.00 | 75.00 | 80.00 | 0 – 100 | 5.94 | 3 | .115 |
| Pain | 62.50 | 67.50 | 67.50 | 77.50 | 0 – 100 | 5.45 | 3 | .142 |
| Social functioning | 62.50 | 75.00 | 75.00 | 75.00 | 0 – 100 | 6.00 | 3 | .113 |
| Health Behaviours |  |  |  |  |  |  |  |  |
| Fruit intake* (CSIRO) | 0.71 | 1.00 | 0.43 | 0.71 |  | 0.87 | 3 | .833 |
| Vegetable intake* (CSIRO) | 2.86 | 2.43 | 1.79 | 2.57 |  | 2.04 | 3 | .565 |
| Bread / cereal intake* (CSIRO) | 3.00 | 2.93 | 3.29 | 2.50 |  | 2.82 | 3 | .421 |
| Meat intake* (CSIRO) | 2.14 | 1.86 | 1.47 | 1.50 |  | 5.49 | 3 | .064 |
| Dairy intake* (CSIRO) | 1.60 | 2.25 | 2.32 | 1.61 |  | 5.10 | 3 | .078 |
| Moderate activity† (GPAQ) | 1.00 | 2.00 | .92 | 2.05 |  | 2.79 | 3 | .425 |
| Vigorous activity† (GPAQ) | 0.00 | 0.00 | 0.00 | 0.00 |  | 6.80 | 3 | .076 |
| Subjective sleep quality (PSQI) | 2.00 | 1.00 | 2.00 | 1.00 | 0 – 3 | 5.94 | 3 | .115 |
| Sleep latency (PSQI) | 1.00 | 1.00 | 1.00 | 1.00 | 0 – 3 | 3.41 | 3 | .332 |
| Sleep duration (PSQI) | 1.00 | 0.00 | 0.00 | 0.00 | 0 – 3 | 5.15 | 3 | .161 |
| Habitual sleep efficiency (PSQI) | 1.00 | 0.00 | 0.00 | 1.00 | 0 – 3 | 4.00 | 3 | .266 |
| Sleep disturbance (PSQI) | 2.00 | 2.00 | 2.00 | 2.00 | 0 – 3 | 0.33 | 3 | .954 |
| Sleep medications (PSQI) | 0.00 | 0.00 | 0.00 | 0.00 | 0 – 3 | 2.52 | 3 | .472 |
| Daytime dysfunction (PSQI) | 2.00 | 1.50 | 2.00 | 1.00 | 0 – 3 | 7.27 | 3 | .064 |
| Alcohol intake (AUDIT) | 4.00 | 3.00 | 3.00 | 3.50 | 0 – 50 | 3.91 | 3 | .272 |
| ** daily servings; † hours per week* | | |  |  |  |  |  |  |

| **eTable 4. Changes in medians over time (outcomes from Wilcoxon Signed-Rank test)** (n=18) | | | | | |
| --- | --- | --- | --- | --- | --- |
|  | **Baseline** | **Post-Intervention** |  |  |  |
|  | ***Median*** | ***Median*** | **Score range** | ***Z*** | ***p*-value** |
| Health Behaviours |  |  |  |  |  |
| Uncontrolled eating (TFEQ-R18) | 57.41 | 40.74 | 0 – 100 | -2.10 | .036* |
| Discretionary food intake*†* (CSIRO) | 6.12 | 3.61 |  | -2.43 | .015* |
| Overall sleep problems (PSQI) | 10.00 | 7.00 | 0 – 21 | -2.36 | .018* |
| ****p*<.001, ***p*<.01, **p*<.05; *† daily servings* | |  |  |  |  |

| **eTable 5. Participants’ subjective ratings* of the program** | |
| --- | --- |
|  | ***M* (*SD*)** |
| Overall quality of program | 4.29 |
| Average session rating | 4.65 |
| Session structure | 4.59 |
| Group dynamic | 4.41 |
| Benefit to mental health | 4.47 |
| Benefit to physical health | 4.06 |
| **scale of 1 – 5; score of 4 reflects rating of “good”)* | |

| **eTable 6. Sample of participants’ qualitative feedback of the program** |
| --- |
| *“The CBT model was a HUGE break for me. I no longer felt weak, stupid and inadequate for being stuck in the cycle. I saw for the first time how crazy and complicated the web is and that it is ok to need help to break free. I actually had hope for the first time in years.”* |
| *“I was hoping to get some help with my head and that an added bonus would be a bit of weight loss. I knew the weight loss was never going to happen while my mental state was so bad.”* |
| *“Hearing that ‘diet’ is a dirty word was another light-bulb moment. I could see why any form of restrictive eating was a temporary fix and why it caused me to obsess over food.”* |
| *“Initially a diary was helping me and also having apps on my phone to help process issues and switch my thinking in the moment helped early on. Mindfulness has been great to learn about and is something I need to make more time for each day.”* |
| *“The program has helped me to identify issues that I didn’t realize were harming me. Knowing I’m not alone has given me a lot of strength.”* |
| *“I know I have a long way to go and I have a lot of work ahead of me but for now I feel I’m getting my life back.”* |
| *“I have dropped a dress size and hope to drop a few more over time. There is no rush. It took years to get here and I know it will take time to leave.”* |

**Supplementary References**

1. Kroenke K, Spitzer RL, Williams JBW. The PHQ-9: Validity of a Brief Depression Severity Measure. *Journal of General Internal Medicine* **16**, 606-613 (2001).

2. Hamilton M. A rating scale for depression. *Journal of neurology, neurosurgery, and psychiatry* **23**, 56-62 (1960).

3. Hamilton M. The assessment of anxiety states by rating. *The British journal of medical psychology* **32**, 50-55 (1959).

4. Rosenberg M. *Society and the adolescent self-image*. Princeton, N.J., Princeton University Press (1965).

5. Cooper PJ, Taylor MJ, Cooper Z, Fairbum CG. The development and validation of the body shape questionnaire. *International Journal of Eating Disorders* **6**, 485-494 (1987).

6. Ware J, Sherbourne CD. THE MOS 36-ITEM SHORT-FORM HEALTH SURVEY (SF-36) .1. CONCEPTUAL-FRAMEWORK AND ITEM SELECTION. *Med Care* **30**, 473-483 (1992).

7. Armstrong T, Bull F. Development of the World Health Organization Global Physical Activity Questionnaire (GPAQ). *Journal of Public Health* **14**, 66-70 (2006).

8. Hendrie G, Baird D, Golley R, Noakes M. The CSIRO Healthy Diet Score: An Online Survey to Estimate Compliance with the Australian Dietary Guidelines. *Nutrients* **9**, 47 (2017).

9. Karlsson J, Persson LO, Sjöström L, Sullivan M. Psychometric properties and factor structure of the Three-Factor Eating Questionnaire (TFEQ) in obese men and women. Results from the Swedish Obese Subjects (SOS) study. *International Journal of Obesity* **24**, 1715 (2000).

10. Svensson M*, et al.* The change in eating behaviors in a Web-based weight loss program: a longitudinal analysis of study completers. *Journal of medical Internet research* **16**, e234 (2014).

11. Saunders John B, Aasland Olaf G, Amundsen A, Grant M. Alcohol consumption and related problems among primary health care patients: WHO Collaborative Project on Early Detection of Persons with Harmful Alcohol Consumption—I. *Addiction* **88**, 349-362 (1993).

12. Buysse DJ, Reynolds CF, Monk TH, Berman SR, Kupfer DJ. The Pittsburgh sleep quality index: A new instrument for psychiatric practice and research. *Psychiatry Research* **28**, 193-213 (1989).
